# Beyond decompression: predictors of cranioplasty failure in pediatric patients – a meta-analysis

**DOI:** 10.1007/s00701-025-06637-x

**Published:** 2025-08-16

**Authors:** Ehab Shabo, Ömer Can Yildiz, Christian Wispel, Muriel Heimann, Sarah-Marie Gallert, Hartmut Vatter, Sevgi Sarikaya-Seiwert

**Affiliations:** 1 Department of Neurosurgery, University Hospital Bonn, Venusberg-Campus 1, 53127 Bonn, Germany; 2 Department of Neurosurgery, Sana Kliniken Duisburg, Zu Den Rehwiesen 9-11, 47055 Duisburg, Germany; 3 Section of Pediatric Neurosurgery, Department of Neurosurgery, University Hospital Bonn, Venusberg-Campus 1, 53127 Bonn, Germany

**Keywords:** Decompressive craniectomy, Cranioplasty, Children, Pediatric cranioplasty, Osteolysis, Meta-analysis

## Abstract

**Background:**

Pediatric cranioplasty following decompressive craniectomy is associated with high complication rates, particularly bone resorption and infection. Unlike adult populations, children face unique anatomical and physiological challenges, and the lack of viable alternatives to autologous bone graft further complicates outcomes. This meta-analysis aims to evaluate the current state of pediatric cranioplasty, identifying key predictors of bone resorption and infection, and assessing outcomes to guide future clinical improvements.

**Methods:**

A systematic search was conducted in MEDLINE/PubMed and Web of Knowledge using combinations of the terms "cranioplasty," "pediatric," "children," and "decompressive craniectomy." Studies were included if they reported quantitative data on outcomes in pediatric populations. Risk of bias were assessed using the ROBINS-I tool.

**Results:**

Seven retrospective case series encompassing 594 patients were analysed. The average age of patients was 8.4 years, with a mean follow-up of 37.8 months. The mean time from craniectomy to cranioplasty was 12.5 weeks. Autologous bone graft was used in 77.6% of cases. The rate of bone resorption requiring reoperation approached 30%, and infection occurred in approximately 10% of patients. Delayed cranioplasty (> 6 weeks), large skull defect area, underlying cerebral contusion, and comminuted fractures significantly predicted bone graft resorption. The use of ventriculoperitoneal shunts, cranial implants, and non-titanium fixation materials were associated with higher infection and resorption rates.

**Conclusion:**

Pediatric cranioplasty carries unacceptably high complication rates for an elective procedure. Early intervention and the use of titanium fixation may reduce the burden of reoperation. Further prospective studies are necessary to establish standardized surgical protocols and explore alternative materials.

**Supplementary Information:**

The online version contains supplementary material available at 10.1007/s00701-025-06637-x.

## Introduction

Decompressive craniectomy (DC) is a critical neurosurgical intervention employed to alleviate elevated intracranial pressure (ICP) in pediatric patients suffering from conditions such as traumatic brain injury (TBI), stroke, or other causes of cerebral edema [3, 5, 15, 18]. While DC can be life-saving, it results in a skull defect that necessitates subsequent cranioplasty (CP) to restore cranial integrity, protect the brain, and improve neurological function [7, 21, 26].

In pediatric populations, CP presents unique challenges distinct from those encountered in adults. The growing skull, higher bone turnover rates, and the potential for cranial deformities complicate the selection of appropriate materials and timing for CP. Autologous bone grafts are commonly used due to their osteogenic potential and compatibility; however, they are associated with a significant risk of bone flap resorption, leading to graft failure and the need for revision surgeries [10, 11, 14, 31].

The incidence of bone flap resorption in pediatric patients varies widely in the literature, with rates reported between 20 and 50% [4, 16]. Several factors have been implicated in increasing the risk of bone flap resorption in children undergoing CP after DC, including younger age, hydrocephalus and the presence of comminuted fractures [4].

Timing of CP is another critical factor influencing outcomes. Early CP, typically defined as occurring within six weeks post-DC, has been associated with significantly lower rates of bone flap resorption compared to delayed procedures [24].

Infection is another prevalent complication following CP, with reported rates ranging from 5% to 12.8% [25]. Significant risk factors for postoperative infections include the presence of ventriculoperitoneal (VP) shunts, gastrostomy tubes, ventilator dependence, and the use of cranial implants [25].

Given the high rates of complications associated with CP in the pediatric population, there is a pressing need to synthesize existing data to navigate clinical decision-making. This meta-analysis aims to evaluate the current state of pediatric CP following DC, identify key predictors of bone resorption and infection, and assess outcomes to guide future clinical improvements.

## Methods

### Reporting guidelines

**Fig. 1:**
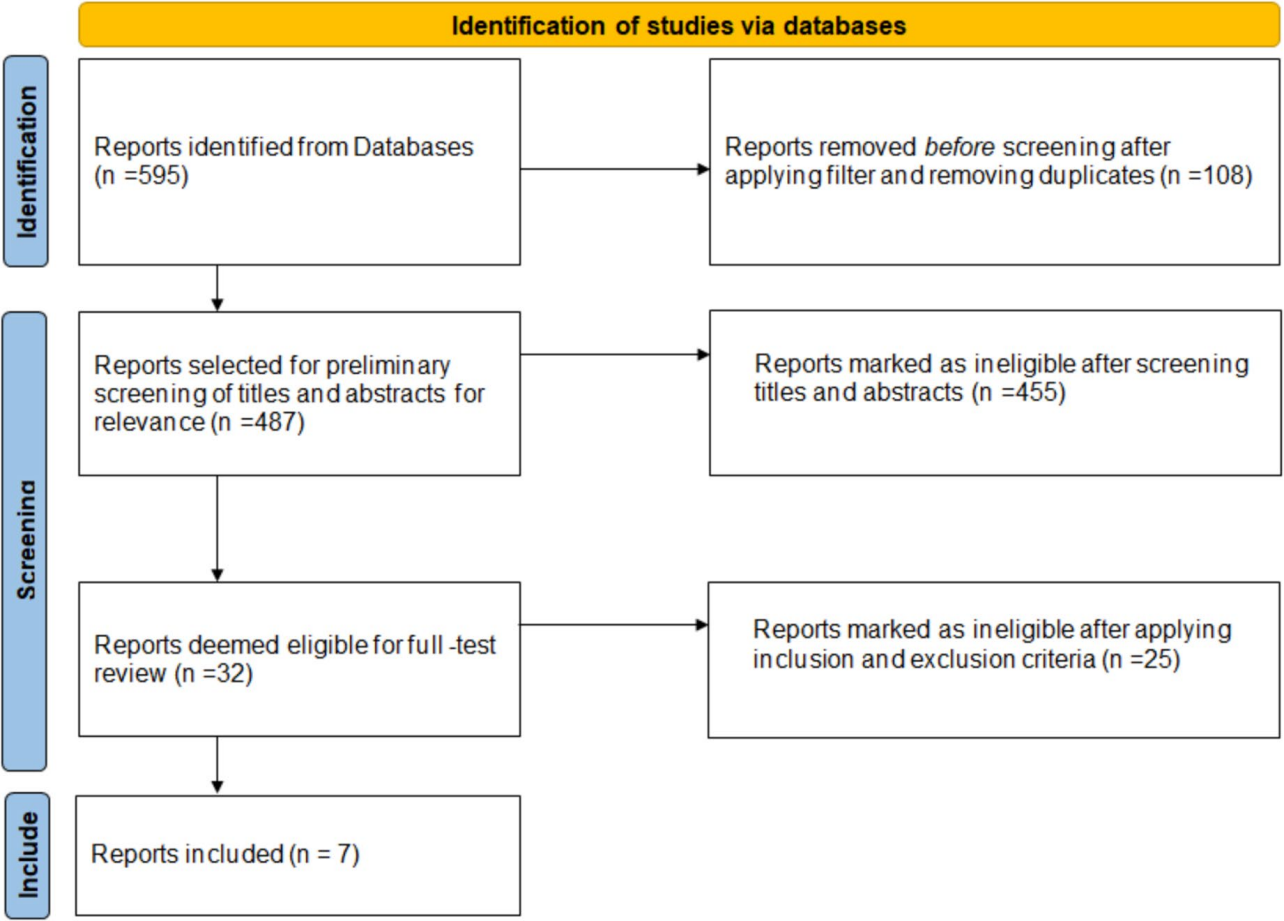
Represents the methodology and study selection of the meta-analysis. PRISMA 2020 flow diagram that describes the methodology of this meta-analysis. (Source: Page MJ, et al. BMJ 2021;372:n71. 10.1136/bmj.n71. This work is licensed under CC BY 4.0. To view a copy of this license, visit)

This meta-analysis was conducted in accordance with the Preferred Reporting Items for Systematic Reviews and Meta-Analyses (PRISMA) 2020 guidelines. The PRISMA checklist was completed and submitted alongside the manuscript to ensure comprehensive and transparent reporting of methods and results. A PRISMA flow diagram (Fig. 1) illustrates the study selection process, including identification, screening, eligibility assessment, and final inclusion of studies. The completed PRISMA 2020 checklist is also provided as a supplementary file.

### Search strategy

A systematic literature search was performed using MEDLINE/PubMed and Web of Knowledge databases covering all publications till December 2024 using the following keywords: ((cranioplasty) AND (pediatric) OR (children)) AND ((cranioplasty) AND (decompression) OR (decompressive) OR (craniectomy)). Filters applied included English language, peer-reviewed articles and full text availability.

### Inclusion and exclusion criteria

Studies were included in this meta-analysis if they were retrospective or prospective in design and provided quantitative data on pediatric patients who underwent cranioplasty following decompressive craniectomy. Studies were excluded if they were individual case reports, lacked quantifiable outcome data, did not clearly specify that the patient population was pediatric, or failed to include sufficient follow-up information.

### Data extraction

The following variables were extracted from eligible reports: Number of patients, age, time interval between decompression and cranioplasty, material used for cranioplasty, incidence of infection and incidence of bone resorption. Overall, seven retrospective case series were included.

### Study selection

The initial database search yielded 595 articles. After applying the mentioned filter strategy and removing duplicates, 487 articles could be identified. Following a preliminary screening of titles and abstracts for relevance, 32 articles were selected for full-text review. After applying the predefined inclusion and exclusion criteria, seven studies were deemed eligible and included in the final analysis. The selection process was conducted in accordance with the Preferred Reporting Items for Systematic Reviews and Meta-Analyses (PRISMA) guidelines.

Methodology is demonstrated as PRISMA 2020 flowchart per the latest guidelines 2020 in Fig. 1.

### Risk-of-bias assessment

The methodological quality and risk of bias for the seven included retrospective case series were assessed using the ROBINS-I tool [28]. This tool evaluates bias across seven domains: confounding, selection of participants, classification of interventions, deviations from intended interventions, missing data, measurement of outcomes, and selection of the reported result. An overall judgment was assigned to each study (low, moderate, serious, or critical risk of bias).

## Results

### Summary of included studies

A total of seven studies met the inclusion criteria and were included in this meta-analysis. Comprehensive details of these studies are summarized in Table 1.

**Table 1:** Demonstrates the eligible studies for the meta-analysis

Citation	Setting	No. of patients	Mean age (years)	Mean follow-up (months)	Mean time between DC and CP (weeks)
Gruber et al., 1988 [12]	retrospective, single-center	17	8.8 (range 1–18)	70.8 (range 48–120)	n.a
Grant et al., 2004 [9]	retrospective, single-center	40	9.3 (range 0.1–18)	57.6 (range 6–72)	20.8 (range 4–68)
Piedra et al., 2012 [24]	retrospective, single-center	61	9.5 (± SD of 5.52)	24 (range 2–124)	9 (range 1–43)
Bowers et al., 2013 [4]	retrospective, single-center	54	6.2 (± SD of 4.7)	37.9 (range 1.5–168)	9.1 (range 1.2–52)
Martin et al., 2014 [19]	retrospective, single-center	27	9.6 (range 0.1–17)	91 (range 18–176)	11.9 (range 2–22)
Waqas et al., 2017 [30]	retrospective, single-center	36	7.1 (± SD of 4.97)	18.2 (± SD of 20.9)	15 (range 4–55)
Roque et al., 2018 [25]	retrospective, multi-center	359	8.4 (± SD of 5.7)	32 (range n.a.)	n.a

*CP:* Cranioplasty;* DC:* Decompressive craniectomy; *n.a.:* Not available; *no.*: Number

### Reporting bias assessment

Due to the limited number of included studies (*n* = 7), a formal statistical assessment of reporting bias, such as funnel plot asymmetry or Egger’s test, was not performed, as these analyses are underpowered in meta-analyses with fewer than 10 studies. Instead, qualitative assessment was conducted by comparing reported outcomes to the stated study objectives and methods in each publication. Confounding emerged as the most frequent source of bias, often due to inadequate adjustment for key variables such as patient age, cranioplasty timing, and comorbidities (e.g., VP shunt presence or pre-existing infection). Selection bias was also evident in some studies with unclear inclusion criteria or incomplete demographic reporting. Additionally, missing data represented another area of concern, especially in older studies that either lacked complete follow-up or failed to report outcomes in a systematic manner. Among the studies analysed, only Roque et al. (2018) demonstrated a low risk of bias across all domains, likely attributable to its multicentre design, larger patient cohort, and more rigorous data collection and reporting practices.

Full domain-specific ratings for each study are summarized in Table 2.

**Table 2:** Summarizes the results of Risk-of-Bias Assessment of the included seven studies

Study	Bias due to confounding	Bias in selection of participants	Bias in classification of interventions	Bias due to deviations from intended interventions	Bias due to missing data	Bias in measurement of outcomes	Bias in selection of the reported result	Overall risk of bias
Gruber et al., 1988	serious	moderate	low	low	serious	moderate	moderate	moderate to serious
Grant et al., 2004	moderate	low	low	low	moderate	low	moderate	low to moderate
Piedra et al., 2012	moderate	low	low	low	moderate	low	low	low to moderate
Bowers et al., 2013	moderate	low	low	low	moderate	low	low	low to moderate
Martin et al., 2014	moderate	low	low	low	moderate	moderate	moderate	moderate
Waqas et al., 2017	serious	moderate	low	low	serious	moderate	moderate	moderate to serious
Roque et al., 2018	low	low	low	low	low	low	low	low

### Demographics of patients and postoperative rate of bone graft resorption and infection

A total of 594 pediatric patients were included, with a mean age of 8.4 years and an average follow-up of 37.8 months (*n* = 533). The mean interval between decompressive craniectomy (DC) and cranioplasty (CP) was 12.5 weeks. Trauma was the predominant indication for DC (95.1%), and unilateral craniectomies (85.1%) were most common among reported locations. The majority of patients (77.6%) received autologous bone grafts, while 22.4% underwent cranioplasty with alloplastic materials. A detailed summary of demographic and procedural data is provided in Table 3.

**Table 3:** Presents a summary of the results

Category	Details
Total number of patients	594
Mean age	8.4 years (range 0.1–18)
Mean follow-up (*n* = 533)	37.8 months (range 1.5–176)
Mean time between decompression and cranioplasty	12.5 weeks (range 1–68)
Indication for craniectomy (*n* = 493)	Trauma: 469
	Spontaneous hemorrhage: 10
	Stroke: 5
	Tumor: 5
	Infection: 3
	Status epilepticus: 1
Craniectomy location (*n* = 195)	Unilateral: 166
	Bifrontal: 2
	Bilateral: 2
Cranioplasty material (*n* = 594)	Autogenous bone flap: 461
	Allograft: 133

**Fig. 2:**
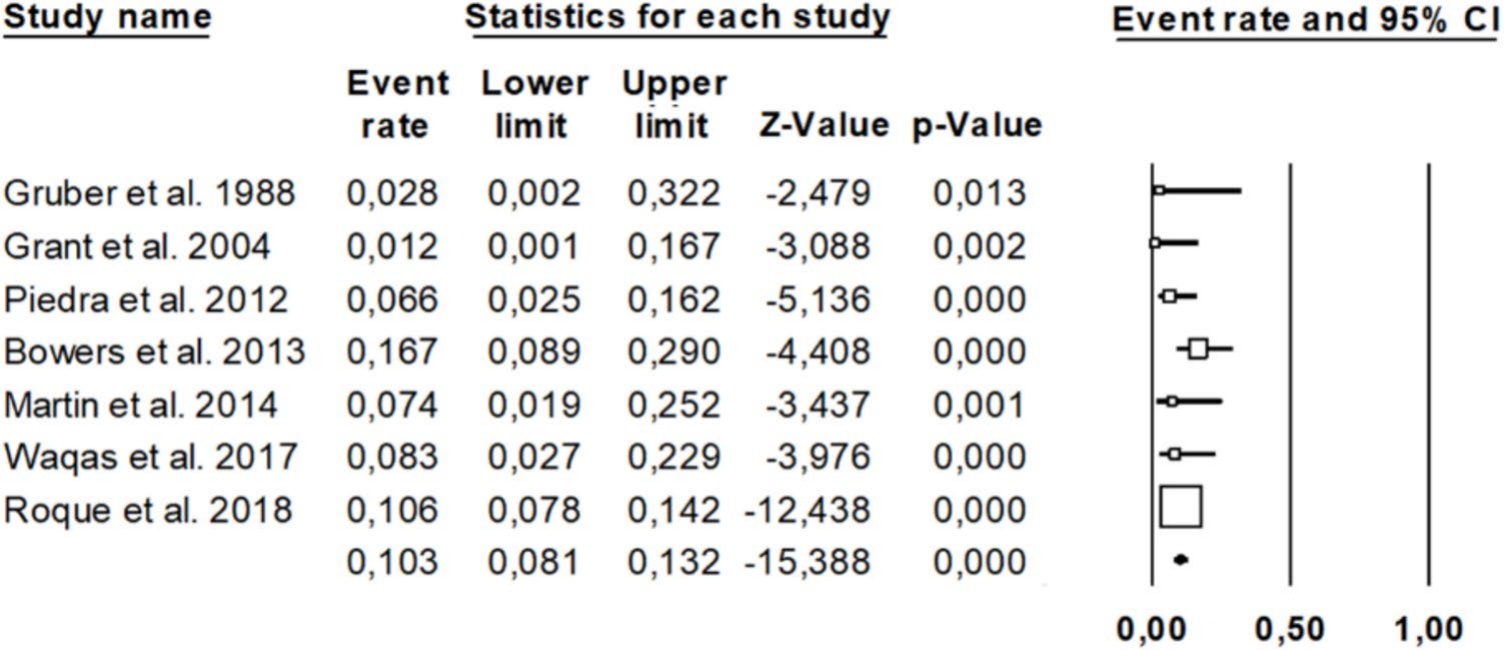
Presents the Forest Plot analysis regarding infection rate after pediatric CP

The pooled infection rate after cranioplasty was 10.3% (95% CI: 8.1%–13.2%) as illustrated in the forest plot (Fig. 2). Individual study event rates ranged from 1.2% (Grant et al. 2004) to 16.7% (Bowers et al. 2013). All included studies demonstrated statistically significant findings (p-values < 0.05). The confidence intervals varied in width, with earlier studies such as Gruber et al. (1988) displaying broader intervals (0.2%–32.2%), suggesting lower precision likely due to smaller sample sizes. In contrast, more recent studies, such as Roque et al. (2018), provided more precise estimates with narrower confidence intervals (7.8%–14.2%) and greater statistical weight. The heterogeneity among studies appears moderate, reflecting some variation in infection rates across different populations or surgical practices. Nonetheless, the findings support a measurable and clinically relevant risk of postoperative infection, emphasizing the need for standardized infection prevention protocols in cranioplasty procedures.

**Fig. 3:**
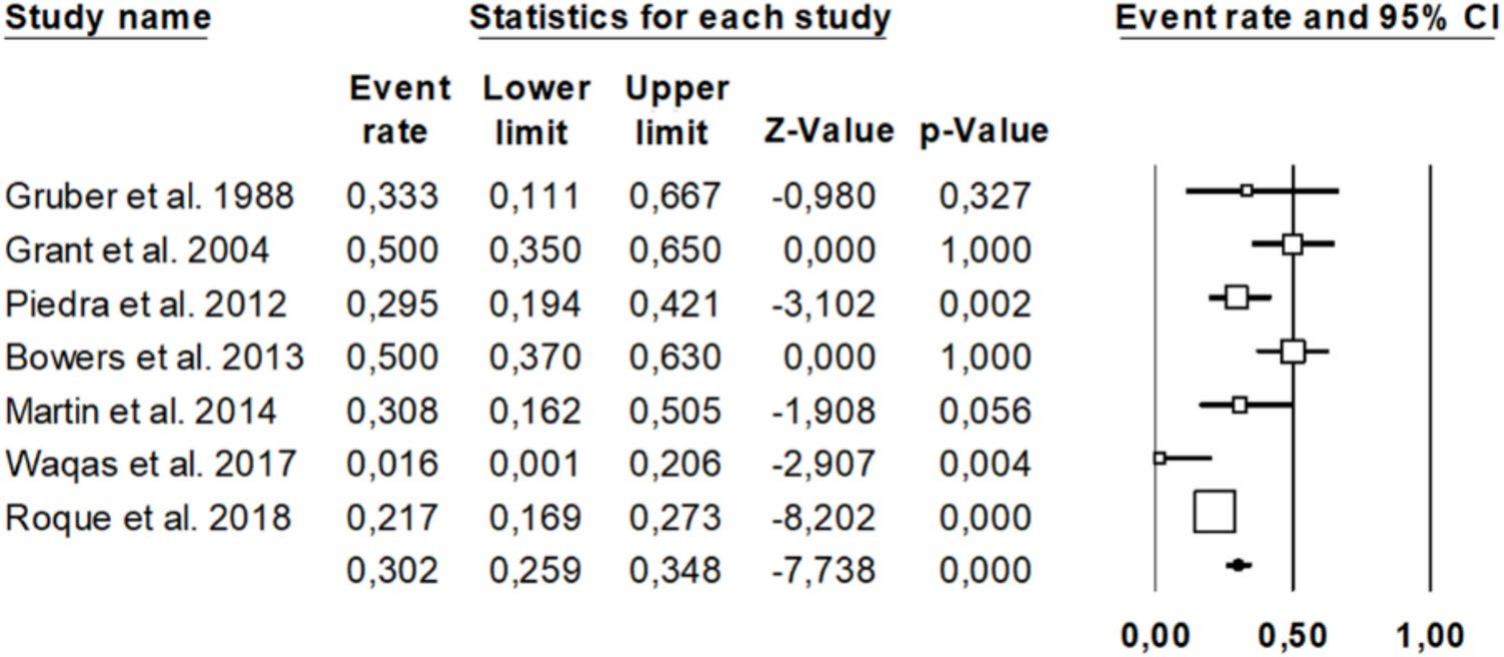
Presents the Forest Plot analysis regarding bone graft resorption rate after pediatric CP

The overall resorption rate was 30.2% (95% CI: 25.9%–34.8%), with considerable variability between studies (range: 1.6% to 50.0%). Some studies with wider confidence intervals yielded non-significant results, likely due to small sample sizes, whereas others (e.g., Roque et al., Piedra et al.) yielded statistically significant findings with narrower confidence intervals, suggesting greater precision and weight in the analysis. Statistical details are presented in the following Forest Plot **(**Fig. 3).

The pooled estimate, represented by the diamond at the bottom of the plot, confirms a substantial and statistically significant risk of bone flap resorption after cranioplasty. These findings highlight the clinical importance of long-term monitoring and material selection in autologous cranioplasty, given the relatively high and variable incidence of resorption reported across studies.

### Predictors of bone graft resorption and infection

Several factors have been identified as significant predictors of bone graft resorption and infection following cranioplasty. Delayed timing of cranioplasty, particularly when performed more than six weeks after the initial craniectomy, was associated with an increased risk of bone graft resorption [24]. Larger skull defect sizes were also correlated with higher resorption rates [9], while comminuted skull fractures and cerebral contusions were linked to poor bone flap outcomes [4]. The choice of fixation material played also a critical role; resorbable plates and sutures were associated with higher failure rates compared to titanium fixation [25]. Additionally, younger patient age and the use of external ventricular drains (EVDs) were independently associated with an increased risk of osteolysis [25]. Regarding postoperative infection, the presence of ventriculoperitoneal (VP) shunts, gastrostomy tubes (G-tubes), ventilator dependence, and cranial implants were all identified as significant risk factors [25].

Regarding non-autologous cranioplasty, multiple studies demonstrate favourable outcomes with synthetic implants in pediatric populations. Gruber et al. [12] reported stable long-term results using methylmethacrylate (MMA) in 10 cases, with no infections, skin breakdown, or graft migration over follow-ups up to 6 years; reoperations were mainly for autologous bone resorption rather than implant failure. Bowers et al. [4] observed a 91.7% success rate in 27 revision cranioplasties with synthetic materials (including PEEK), significantly outperforming autologous grafts, which had a 28.5% failure rate. Grant et al. [9] noted low postoperative infection rates after revision with synthetic implants (MMA, hydroxyapatite, titanium mesh), with most revised implants remaining complication-free. Martin et al. [19] reported high osteolysis rates (66.7%) with autologous bone and frequent reoperations, while synthetic materials provided more reliable outcomes. Waqas et al. [30] found low complication rates (infection at 8.3%) in 36 pediatric cases using MMA or titanium mesh, with no significant impact of implant type on complications or cosmetic satisfaction. Rocque et al. [25] highlighted higher resorption rates in resorbable/autologous bone (21.7%) versus lower rates with synthetic fixation such as titanium plates.

In summary, non-autologous materials (MMA, HA, PEEK, titanium mesh) offer superior structural stability, reduced resorption, and acceptable cosmetic outcomes, with comparable or lower infection and reoperation rates relative to autologous grafts, particularly in cases of prior bone flap failure. However, subgroup-level statistical outcome data for these materials (e.g., infection, cosmetic failure, re-operation) were not consistently reported across included studies.

While Waqas et al. and Grant et al. did not report storage methods of the autologous bone grafts, other included studies reported that cryopreservation of autologous bone flaps, typically at temperatures ranging from − 25 °C to − 85 °C, did not significantly affect outcomes such as bone resorption or infection. Moreover, although early re-implantation is often preferred to minimize potential osseous damage, available evidence suggests that standard cryopreservation practices do not negatively impact graft viability.

## Discussion

This meta-analysis highlights the persistent challenges and substantial complication rates associated with pediatric CP following DC. With an overall bone flap resorption rate approaching 30% and infection rates exceeding 10%, the findings underscore the complexity and risk profile of CP in children. These risks appear disproportionately high for what is typically considered an elective reconstructive procedure.

It is also important to recognize that pediatric patients under the age of 2 years, typically characterized by open fontanelles and unfused cranial sutures, represent a distinct subgroup with unique anatomical and surgical considerations. This physiologic difference may affect both the healing response and the risk of complications following cranioplasty. However, most of the historical studies included in our meta-analysis did not report age-stratified outcomes for this cohort, limiting the feasibility of a meaningful subgroup analysis. A case review by Behbahani et al. [2] has specifically underscored the paucity of outcome data in this population and highlighted the distinct challenges in performing cranioplasty in children under 2 years of age following decompressive craniectomy (DC). Moreover, this age group is increasingly targeted by novel surgical techniques, such as the Decompressive Craniotomy in Split Technique (DCST) described by Sarikaya et al. [27], which preserves the bone flap in situ and enables postoperative monitoring through the fontanelle via ultrasound. These innovations not only demonstrate the adaptability of the infant skull but also have the potential to spare patients from future cranioplasty altogether. Given these emerging paradigms and the limitations of current data, we chose to focus this meta-analysis on the broader pediatric population and recommend that future studies investigate this younger subgroup separately, with appropriately stratified outcome reporting.

The high incidence of bone graft resorption is a central concern, with multiple studies confirming this as a common postoperative complication in pediatric populations [4, 16]. The mean resorption rate of 30.2% aligns with prior reports, although individual study rates ranged widely. Importantly, our findings emphasize that delayed CP, particularly beyond six weeks post-DC, is significantly associated with increased risk of resorption. This reinforces growing evidence that early cranioplasty may confer a protective effect against osteolysis, possibly by re-establishing normal cranial physiology and minimizing inflammatory remodelling processes. Furthermore, large skull defects, comminuted fractures, and underlying cerebral contusions emerged as key predictors of bone flap failure, likely reflecting greater initial injury severity and impaired graft viability.

The choice of material for cranioplasty also significantly influenced outcomes. Autologous bone remains the preferred graft type due to its biological compatibility and cost-effectiveness [11, 14, 31]. However, the high osteolysis rates suggest limitations of autograft use in children, whose active bone remodelling and growth processes may predispose them to resorption. In contrast, alloplastic materials, while potentially reducing resorption, carry their own risks, particularly for infection and implant failure. The data from this analysis suggest that titanium fixation may offer a more favourable balance, being associated with lower complication rates compared to resorbable fixation systems.

Infection remains the second major complication identified in this review. The pooled infection rate of 10.3%, although consistent with existing literature [25], remains clinically significant, especially considering the vulnerability of pediatric patients to prolonged hospitalizations and neurodevelopmental impacts from repeat surgeries. Risk factors such as VP shunts, G-tubes, and ventilator dependence suggest that infection risk is strongly associated with underlying medical complexity and possibly immune-compromised states. These associations emphasize the importance of careful perioperative planning and infection control protocols, especially in medically complex children.

In comparison with adult cranioplasty data, where infection rates typically lower and range from 1–13% and bone resorption is relatively less common and varies between 2 and 30% [1, 6, 8, 13, 17, 20, 22, 23, 29], our findings of a 10.3% infection rate and 30.2% resorption in pediatric patients reflect fundamentally different clinical challenges. This difference likely stems from age-specific factors such as higher osteogenic turnover and active cranial growth in children. Other pediatric reviews have similarly documented high complication rates but were limited by smaller sample sizes and less extensive bias assessment [1, 14, 32]. By incorporating data from nearly 600 children and using a systematic risk-of-bias approach, our study provides more precise complication estimates and affirms pediatric-specific risk profiles, laying groundwork for age-adapted clinical guidelines and future research.

Methodologically, this review was limited by the retrospective design and overall approximately moderate risk of bias in most included studies. The absence of consistent definitions for timing, outcome measurement, and confounding adjustment limit the generalizability of the pooled results. Only one study (Roque et al., 2018) demonstrated low risk of bias across all domains, highlighting a clear need for more prospective, standardized studies in this field. Additionally, the mean follow-up duration of 37.8 months must be acknowledged as a limitation, particularly in the context of alloplastic cranioplasty materials, where late-onset complications may emerge beyond this period. The limited follow-up is a reflection of the retrospective nature and methodological constraints of the included studies. As such, our findings may underestimate the true incidence of late complications. Future prospective, longitudinal studies with extended follow-up are essential to better capture the durability and long-term safety of cranioplasty materials, especially in growing pediatric patients.

Despite these limitations, this meta-analysis offers clinically meaningful insights. First, early cranioplasty should be considered more routinely when feasible, given its apparent protective effect against bone resorption. Second, the routine use of titanium-based fixation systems may reduce both infection and resorption rates. Finally, the high variability across centres in surgical technique and material use underscores the lack of consensus or standardized guidelines in pediatric cranioplasty, an area that must be addressed through multicentre trials and collaborative registries.

Overall, while this meta-analysis may not introduce novel individual risk factors, it represents the largest pooled cohort to date examining pediatric cranioplasty following decompressive craniectomy. By synthesizing data from nearly 600 patients across seven studies, it offers enhanced statistical precision in estimating key complication rates, specifically bone flap resorption and infection, compared to smaller, single-center reports. This increased sample size allows for narrower confidence intervals and more robust, clinically applicable findings. Furthermore, our analysis provides valuable insight into the influence of patient- and procedure-specific variables (e.g., age, timing of cranioplasty, presence of VP shunts, defect size, and fixation materials), which have previously been inconsistently reported. By systematically assessing risk of bias across studies, our work also helps contextualize the strength of available evidence. These contributions strengthen the foundation for multicenter standardization and guide the design of future prospective research in this vulnerable patient population.

## Conclusion

Pediatric cranioplasty following decompressive craniectomy continues to present a substantial complication burden, particularly bone flap resorption and infection. While autologous bone remains the mainstay of reconstruction, its limitations in the pediatric population demand reconsideration of timing and materials. Early cranioplasty, titanium fixation, and individualized risk stratification based on comorbidities may mitigate adverse outcomes. Ultimately, well-designed prospective studies are needed to establish standardized protocols and improve long-term outcomes in this vulnerable population.

## Supplementary Information

Below is the link to the electronic supplementary material.

ESM1 (DOCX 266 KB)

ESM2 (DOCX 278 KB)

## Data Availability

No datasets were generated or analysed during the current study.

## Abbreviations

CP: Cranioplasty
DC: Decompressive craniectomy
TBI: Traumatic brain injury

## Declarations

### Registration and protocol

Not applicable, not registered.

### Current state

The Manuscript is not under consideration for publication anywhere.
